# Profile of 1-month training load in male and female football and futsal players

**DOI:** 10.1186/s40064-016-2327-x

**Published:** 2016-05-23

**Authors:** Filipe Manuel Clemente, Pantelis Theodoros Nikolaidis

**Affiliations:** Escola Superior de Desporto e Lazer, Instituto Politécnico de Viana do Castelo, Complexo Desportivo e Lazer de Melgaço - Monte Prado, 4960-320 Melgaço, Portugal; Department of Physical and Cultural Education, Hellenic Army Academy, Athens, Greece

**Keywords:** Internal load, Training microcycle, Gender, Futsal, Soccer, Heart rate

## Abstract

The aim of this study was to analyse the variance of training load between male and female football and futsal players. The statistical analysis tested the variance between gender and type of sport during training sessions. Fifty-nine male and female amateur football and futsal amateur players were monitored during 48 training sessions. The heart rate (HR) responses and the percentage of time spent in zones of intensity were analysed during training sessions. Differences were found in football between the gender and the dependent variables of %HRmax (*p* value = 0.001; *η*^2^ = 0.042; *minimum effect*), %time in Z2 (*p* value = 0.001; *η*^2^ = 0.054; *minimum effect*), %time in Z4 (*pvalue* = 0.001; *η*^2^ = 0.031; *minimum effect*) and %time in Z5 (*p value* = 0.001; *η*^2^ = 0.053; *minimum effect*). The analysis in male players revealed differences between football and futsal in %HRmax (*p value* = 0.001; *η*^2^ = 0.172; *minimum effect*). Similar results were found in female category (*p* value = 0.001; *η*^2^ = 0.040; *minimum effect*). In this study it was possible to verify that female players spent more time in high intensity zones and that futsal training sessions are more intense than football sessions. Based on such results, coaches and fitness trainers may identify the physiological characteristics of training load imposed to different sports and genders and optimize the training plan for specific categories.

## Background

Prescribing exercise intensity is one of the main concerns of coaches and fitness trainers when they design a training program. In team sports such as football and futsal exercise intensity might vary depending on training content (e.g. conditioning, tactics). Football and futsal should be considered as related team sports, since many coaches, trainers and athletes get involved in both. Therefore, it would be of great practical importance to be aware of differences in training intensity between these sports. As long as both sexes participate in both sports, coaches should be aware of possible sex differences with regards to training intensity.

Comparative studies on football and futsal players have focused in anthropometric (Burdukiewicz et al. 2014) and physiological characteristics such as aerobic capacity (Baroni et al. 2011), muscle power (da Silva et al. 2012) and agility (Benvenuti et al. 2010; Milanovic et al. 2011). Compared to their football counterparts, futsal players were shorter, lighter and fatter (Burdukiewicz et al. 2014). In a study on professional male athletes, futsal players had similar maximal oxygen uptake (VO_2max_) as football players (~55 mL/kg/min^−1^) but lower anaerobic threshold (Leal Junior et al. 2006). This trend, i.e. similar VO_2max_ but lower anaerobic threshold in futsal, was confirmed later in another study (Baroni et al. 2011). With regards to muscle power, assessed by countermovement jump, male futsal and football players had similar performance (da Silva et al. 2012). A comparison on agility of female regional athletes showed that futsal players had better performance in a reactive visual stimuli test and decision making time than in football players (Benvenuti et al. 2010). On the other hand, football and futsal male players had similar agility as this parameter was tested by slalom tests with and without ball and sprints with various changes of direction (Milanović et al. 2011).

In addition to these anthropometric and physiological differences between football and futsal, certain differences have been observed within each sport with regards to sex. For instance, male football players were taller and heavier, but with similar body mass index, and with lower body fat percentage than their female counterparts (Nikolaidis 2010). This finding was confirmed by another study, which additionally showed that males had better performance in upper and lower limb strength, sprint, change of direction and aerobic capacity than female football players (Ramírez-Campillo et al. 2015). Female football players had lower aerobic capacity, i.e. maximal aerobic speed (Baumgart et al. 2014), and jumping ability—squat jump and countermovement jump—than males (Castagna and Castellini 2013).

The training load imposed by coaches to improve fitness parameters of players have been mostly researched in elite male soccer players (Casamichana et al. 2013; Coutinho et al. 2015; Impellizzeri et al. 2004). The study conducted by Impellizzeri et al. (2004) that used a Foster’s RPE based approach revealed that the greater training load occurred in Monday session (second day after match) and the training load progressively decreased over the week. A more recent study using GPS monitoring in elite soccer players revealed that in U15 the technical and elementary tactical skills are similar in post-match and middle week training sessions, the middle week sessions are more intense in U17 players and that the greater value of intensity in post-match are associated with prescription to substitute players in the U19 players (Coutinho et al. 2015).

Despite of these findings in elite male soccer players, the research it has not focused in female gender and in similar team sports such as futsal (indoor-football). Moreover, most of the existing literature about research of differences between football and futsal, and between female and male athletes has concentrated on anthropometric and physiological characteristics, and less information was available about differences in training parameters such as heart rate (HR) responses to various training contents (Condessa et al. 2015; Wrigley et al. 2012). Therefore, the aim of the present study was to examine the effect of sport (football vs. futsal) and sex on exercise intensity during various training content.

## Methods

### Subjects

The subjects of this study were 20-male amateur football players, 18-female amateur football players, 11-male amateur futsal players, and 10-female amateur futsal players (see Table 1). These players belong to four different football and futsal teams competing in Portuguese championships. The goalkeepers participated in the training sessions but were excluded from all data analysis. Before the commencement of the study, all subjects received written and verbal explanations of the procedure involved in the study informing them of all risks and benefits associated with participation, and written informed consent was signed from all of them. The study followed the recommendations of Declaration of Helsinki for the study in humans and follow Ethics, consent and permissions practices.

**Table 1:** Description of players’ subsamples

	Male footballers (*n* = 20)	Female footballers (*n* = 18)	Male futsal players (*n* = 11)	Female futsal players (*n* = 10)
Age (years)	20.4 ± 3.12	19.3 ± 2.22	27.2 ± 4.90	23.3 ± 3.50
Height (m)	180.0 ± 6.00	160.1 ± 5.36	179.0 ± 5.11	162.2 ± 7.18
Weight (kg)	73.4 ± 7.10	54.21 ± 9.46	77.4 ± 6.7	58.1 ± 10.30
Years of Practice (*n*)	11.2 ± 3.1	7.6 ± 2.8	13.9 ± 5.6	5.3 ± 2.2
HRmax (bpm)	193.2 ± 6.4	194.9 ± 5.9	188.7 ± 8.1	191.1 ± 7.6
Yo–YoIR1 (m)	2101.2 ± 506.6	1198.4 ± 189.3	2309.4 ± 238.7	1271.8 ± 231.1
Training Sessions (*n*)	12	12	12	12
Weeks (*n*)	4	4	4	4

### Experimental approach

In the present research, an objective descriptive design was used and a real evaluation of the training load experienced by each player was monitored according to coach prescription without any external intervention as carried out in previous studies (Manzi et al. 2010). The data for this study was collected from four amateur football and futsal teams over a 4-weeks training period. HR was monitored during each training session. The internal load was determined using the %HRmax and the time per zones of intensity. The evolution of the training load profile of these players was examined during the in-season part of competitive season (4 months after the beginning of the season and 4 months before the end of the season). The typical weekly training load profile of the players was assessed during typical weekly microcycle with 1-competition fixtures.

### Procedures

The amateur players (soccer and futsal) had three training sessions per week. In the case of football, it was verified a range of temperature between 12° and 17° and relative humidity 49–57 %. The training sessions in male football players occurred from 20.00 h to 22.00 h and in female players from 2030 to 2230 hours in turf fields. In the case of male futsal players the training sessions occurred between 2030 and 2230 hours and in female futsal players from 2130 and 2300 hours. In futsal players the training occurred with a range of temperature between 18° and 21° and relative humidity 37–41 %. From 1 week to another, it was ensured the same conditions of training to avoid the circadian variations. All teams trained on official field dimension of their sport. None of the participants participated in other physical activity.

The duration of a session was recorded from the start to the end of the session, including recovery periods. Training session duration ranged 80–100 min. All the training sessions examined were prescribed by the head coach and fitness trainer of the team with no external training advice. During this period of the season, most of physical conditioning was performed using drill-based tasks. The main focus on competition lead to the prominence in participated in game-like situations with high tactical demands. It was possible to observe that tasks were designed to develop team tactical organization and interactions between players’ positions. In the case of footballers these tasks usually included players’ superiority/inferiority (GR + 6 × 7; GR + 7 × 4 + GR) to foster the emergence of the targeted behaviour. Similar strategies were observed in futsal teams.

The HR data were recorded via Bluetooth technology (Polar Team App, Polar Electro Oy, Finland) in all training sessions. The HR results were grouped into five different zones of %HRmax: zone 1 [Z1] (50–60 % HRmax), zone 2 [Z2] (60–70 % HRmax), zone 3 [Z3] (70–80 % HRmax), zone 4 [Z4] (80–90 % HRmax), and zone 5 [Z5] (≥90 % HRmax). To measure the players’ HRmax the Yo–Yo Intermittent Recovery Test level 1 was performed (Montgomery et al. 2010).

### Statistical analyses

The data were presented as mean (M) ± standard deviation (s). A two-way MANOVA (followed by one-way ANOVA per factor) was performed to identify differences in %HRmax average, %time spent in Z1, Z2, Z3, Z4 and Z5 intensity zones according to the different genders and type of sport. Pairwise differences and post hoc comparisons were assessed with the Bonferroni post hoc test. Effect size (ES) was presented as $\eta^{2}$ and interpreted using the follow criteria (Ferguson 2009): no effect ($\eta^{2}$ < 0.04), minimum effect (0.04 < $\eta^{2}$ < 0.25), moderate effect (0.25 < $\eta^{2}$ < 0.64) and strong effect ($\eta^{2}$ > 0.64) (Ferguson 2009). All data sets were tested for each statistical technique and corresponding assumptions and performed using SPSS software (version 23.0, Chicago, Illinois, USA). Statistical significance was set at 5 %.

## Results

The two-way MANOVA revealed that the gender (*p* value = 0.001; $\eta^{2}$ = 0.082; *minimum effect*) and type of sport (*p* value = 0.001; $\eta^{2}$ = 0.215; *moderate effect*) had significant main effects on the HR variables. There was significant interaction (Pillai’s Trace = 0.031; *p* = 0.001; $\eta_{p}^{2}$ = 0.031; *no effect*) between the gender and the type of sport. As previously indicated in the statistical procedures, a two-way ANOVA was conducted for each dependent variable after the confirmation of the interaction (O’Donoghue 2012, p. 243). Interactions between factors were found in %HRmax (*p* value = 0.001; $\eta^{2}$ = 0.008; *minimum effect*), %time in Z2 (*p* value = 0.001; $\eta^{2}$ = 0.009; *minimum effect*) and %time in Z4 (*p* value = 0.001; $\eta^{2}$ = 0.009; *minimum effect*). No interactions were found between factors in %time in Z1 (*p* value = 0.355; $\eta^{2}$ = 0.001; *no effect*), %time in Z3 (*p* value = 0.304; $\eta^{2}$ = 0.001; *no effect*) and %time in Z5 (*p* value = 0.558; $\eta^{2}$ = 0.001; *no effect*).

The descriptive statistics can be found in the following Table 2. Differences were found in football between the gender and the dependent variables of %HRmax (*p* value = 0.001; $\eta^{2}$ = 0.042; *minimum effect*), %time in Z2 (*p* value = 0.001; $\eta^{2}$ = 0.054; *minimum effect*), %time in Z4 (*p* value = 0.001; $\eta^{2}$ = 0.031; *minimum effect*) and %time in Z5 (*p* value = 0.001; $\eta^{2}$ = 0.053; *minimum effect*). Male football players spent more time in Z2 (52.96 %) and Z3 (0.75 %) than female players. Nevertheless, female football players spent more time in Z1 (8.08 %), Z4 (90.67 %) and Z5 (361.38 %), moreover achieve statistically greater values of %HRmax (9.98 %).

**Table 2:** Descriptive HR values of the studied participants and the comparisons between genders and type of sport

	Male	Female	*p* value	$\eta^{2}$
*Football*				
%HRmax CI (95 %) $\eta^{2}$	63.55 (9.52)* [62.91–64.19] 0.172—minimum effect	69.89 (12.76)* [67.90–71.89] 0.40—minimum effect	0.001	0.042—minimum effect
%time in Z1 CI (95 %) $\eta^{2}$	25.49 (21.26)* [24.17–26.80] 0.097—minimum effect	27.55 (37.37)* [23.37–31.73] 0.088—minimum effect	0.367	0.001—no effect
%time in Z2 CI (95 %) $\eta^{2}$	24.68 (18.51)* [23.37–25.99] 0.005—no effect	11.61 (14.69)* [8.52–14.70] 0.022—no effect	0.001	0.054—minimum effect
%time in Z3 CI (95 %) $\eta^{2}$	17.37 (16.20)* [16.13–18.60] 0.047—minimum effect	15.85 (17.31)* [12.30–19.39] 0.021—no effect	0.332	0.001—no effect
%time in Z4 CI (95 %) $\eta^{2}$	8.79 (13.82)* [7.63–9.94] 0.187—minimum effect	16.76 (19.40)* [12.64–20.87] 0.030—no effect	0.001	0.031—no effect
%time in Z5 CI (95 %) $\eta^{2}$	1.45 (6.08)* [0.74–2.16] 0.076—minimum effect	6.69 (13.17)* [2.98–10.41] 0.026—no effect	0.001	0.053—minimum effect
*Futsal*				
%HRmax CI (95 %) $\eta^{2}$	73.43 (9.98)* [72.38–74.48] 0.172—minimum effect	75.29 (10.82)* [74.16–76.42] 0.40—minimum effect	0.018	0.008—no effect
%time in Z1 CI (95 %) $\eta^{2}$	10.83 (15.61)* [8.66–13.00] 0.097—minimum effect	10.50 (17.11)* [8.13–12.87] 0.088—minimum effect	0.789	001—no effect
%time in Z2 CI (95 %) $\eta^{2}$	21.49 (22.87)* [19.34–23.65] 0.005—no effect	17.78 (18.32)* [16.03–19.54] 0.022—no effect	0.017	0.008—no effect
%time in Z3 CI (95 %) $\eta^{2}$	26.63 (24.02)* [24.60–28.66] 0.047—minimum effect	22.73 (20.89)* [20.72–24.74] 0.021—no effect	0.021	008—no effect
%time in Z4 CI (95 %) $\eta^{2}$	27.60 (24.69)* [25.70–29.50] 0.187—minimum effect	26.34 (24.44)* [24.01–28.68] 0.030—no effect	0.498	0.001—no effect
%time in Z5 CI (95 %) $\eta^{2}$	8.34 (17.96)* [7.18–9.51] 0.076—minimum effect	14.63 (23.03)* [12.52–16.74] 0.026—no effect	0.001	0.022—no effect

* Statistically differences between football and futsal for a *p* value <0.05

**Fig. 1:**
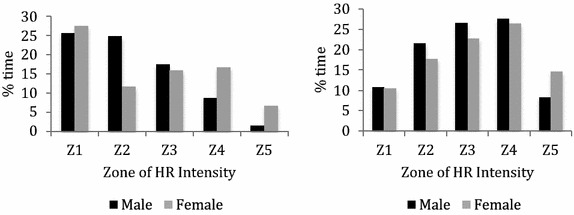
Percentage of time per HR zone in soccer (*left*) and futsal (*right*)

In the analysis carried out in futsal, it were found differences between gender in %HRmax (*p* value = 0.018; $\eta^{2}$ = 0.008; *no effect*), %time in Z2 (*p* value = 0.017; $\eta^{2}$ = 0.008; *no effect*), %time in Z3 (*p* value = 0.021; $\eta^{2}$ = 0.008; *no effect*) and %time in Z5 (*p* value = 0.001; $\eta^{2}$ = 0.022; *no effect*). Male futsal players spent more time in Z1 (3.05 %), Z2 (17.26 %), Z3 (14.65 %) and Z4 (4.57 %) than female players. On the contrary, female futsal players achieved greater %HRmax (2.53 %) and time spent in Z5 (75.42 %) than male players. The comparison between genders can be observed in Fig. 1.

The analysis in male players revealed differences between football and futsal in %HRmax (*p* value = 0.001; $\eta^{2}$ = 0.172; *minimum effect*), %time in Z1 (*p* value = 0.001; $\eta^{2}$ = 0.097; *minimum effect*), %time in Z2 (*p* value = 0.00 $\eta^{2}$ = 0.005; *no effect*), %time in Z3 (*p* value = 0.001; $\eta^{2}$ = 0.047; *minimum effect*), %time in Z4 (*p* value = 0.001; $\eta^{2}$ = 0.187; *minimum effect*) and %time in Z5 (*p* value = 0.001; $\eta^{2}$ = 0.076; *minimum effect*). In futsal it were achieved greater %HRmax values (15.55 %) and time spent in Z3 (53.31 %), Z4 (213.99 %) and Z5 (475. %) than football. On the other hand, in football it were spent more time in Z1 (57.51 %) and Z2 (12.93 %) than in futsal.

In female players it were found differences between football and futsal in %HRmax (*p* value = 0.001; $\eta^{2}$ = 0.040; *minimum effect*), %time in Z1 (*p* value = 0.001; $\eta^{2}$ = 0.088; *minimum effect*), %time in Z2 (*p* value = 0.001; $\eta^{2}$ = 0.022; *no effect*), %time in Z3 (*p* value = 0.001; $\eta^{2}$ = 0.021; *no effect*), %time in Z4 (*p* value = 0.001; $\eta^{2}$ = 0.030; *no effect*) and %time in Z5 (*p* value = 0.001; $\eta^{2}$ = 0.026; *no effect*). It was found greater values of %HRmax (7.73 %), %time in Z2 (53.14 %), %time in Z3 (43.41 %), %time in Z4 (57.16 %) and %time in Z5 (118.68 %) in futsal than in football. Only in %time of Z1 (61.89 %) it was found greater values in football than in futsal.

## Discussion

The aim of this study was to analyse the variance of HR intensities during training sessions in football and futsal for both male and female players. Some research have been working in characterize the training load profile of elite male soccer players, as far we know (Coutinho et al. 2015; Ian Lambert and Borresen 2010; Impellizzeri et al. 2004). Nevertheless, a lack of research in female gender and in emerging and similar team sport (futsal) led us to analyse both sports and both genders. Statistical differences were found in HR responses between football and futsal. Specifically, it was observed that during training sessions futsal players spent more time in high intensity zones of HR and football players spent more time in moderate intensities. Moreover, it was also found differences in HR responses during training sessions between male and female players. In this case, it was found that female football players spent more time in great intensities than male football players. On the other hand, in futsal no differences were found between genders in the highest HR zones, only revealing differences in moderate intensities.

The results of our study revealed that female football players spent more time in Z4 and Z5 of intensity, thus suggesting that the training may be constrained by the fitness level of players. In a study conducted in football players from the Danish women Premier League it was verified that average HR during the game was 86 ± 1 % of maximal HR with no differences between halves (Krustrup et al. 2010). The values found in male football players are also similar (~83 to ~87 %HRmax) (Impellizzeri et al. 2006; Stroyer et al. 2004). In the case of VO2max, some studies suggest that elite female football players may reaches ~54 mL/kg/min^−1^ what is smaller than the regular values of ~66 mL/kg/min^−1^ in male elite players (Bangsbo 2003; Ingebrigtsen et al. 2011; Krustrup et al. 2005; Stølen et al. 2005). A research in female football revealed values of 5.1 mmol/L achieved during the match (Krustrup et al. 2010) what is smaller than the values achieved by male players 6–8 mmol/L (Stølen et al. 2005). The evidences about the VO_2max_ and the level of intensity suggest that female players can be highly influenced by demanding tasks, thus increasing the time spent in vigorous activity. On the contrary, the greater capacity of male players may reduce the possibility to achieve high levels of intensity during training because to achieve that it is required a greater demanding from the tasks. It was also found that female football players spent more time in Z1 than male players. This may be associated with the great intensity that they achieve, thus requiring more periods of low intensity to actively recover between demanding tasks. On the other hand, the training sessions of male players may suggest that coaches opt to a moderate-to-vigorous intensity with smaller periods of very-low or very-high intensities, thus more in continuous regimen.

The statistical analysis that compared the training load between genders in futsal sessions only revealed differences in the average of HRmax, %time spent in Z2 (60–70 %HRmax) and 3 (71–80 %HRmax). The greater average of HRmax it was achieved in female players and the greater percentage of time spent in Z2 and Z3 were found in male players. A study that monitored the training intensities of male futsal players revealed HR intensities between 60 and 70 % of HRmax in Goalkeepers, 71–90 % in forward (pivot) and 81–100 % in wings (Arins and Silva 2007). Nevertheless, no similar study was conducted in female futsal players. Nevertheless, the physiological characterization of female futsal players is similar with findings in football, thus the lower aerobic capacity of female players contribute to reach greater intensities than male players.

The present study also compared the training load imposed for football and futsal. The analysis of variance revealed that futsal had greater intensities during training sessions. Particularly, during futsal sessions it was spent more time in Z3, Z4 and Z5 of HR intensities, thus revealing the greater anaerobic participation. In fact, the physiological characterization to the futsal matches revealed that is a multiple-sprints sport in which there are more high-intensity phases than in football and other intermittent sports (Barbero-Alvarez et al. 2008). For that reason, it is expected that the futsal training sessions follow the demands of the sport, thus keeping greater intensities than football. The greater intensity achieved can be also characterized by the greater amount of short-and-fast activities, such as accelerations and decelerations. A study that analysed the agility and reaction time between female football and futsal players revealed that futsal players were significantly faster in reactive agility and in decision-making (Benvenuti et al. 2010). Despite the greater intermittence of effort and the greater tendency to perform sprints and quick actions, the aerobic capacity is also developed in futsal. A study that compared futsal training with other team sports activities in 8-weeks training programme in VO_2max_ of female college students revealed that futsal increased the maximum VO_2max_ and maximum power in comparison with the control group (Karahan 2012).

The findings of the present study were limited by the lack of time-motion analysis. This specific type of analysis would be useful to justify the different intensities achieved between genders and sports. Moreover, the analysis to the specific type of tasks would be an important contribution to identify the reasons to the different intensities reached between genders for the same sport. For that reasons, would be important that future research identify the time and type of tasks imposed by coaches between genders and also use some tracking systems (such as Global Positioning System) to identify the time-motion profiles of players during training sessions.

Despite of study limitations, it is important to highlight that this study is pioneer of having compared the training load between gender and similar team sports, thus providing an important contribution to coaches and sport scientists with special interest in metabolic impact of futsal and football training. Moreover, the data provided from this study may help to adopt the best periodization or the specific sport and identify possible lines of research in training load analysis made in intermittent sports.

## Practical implications

This study revealed that futsal training induces a moderate-to-vigorous (between 60 and 90 % of HRmax) effort during ~75 % of the time. Such analysis may suggest that a high-intensity aerobic training and lactate threshold is usually the target of workout. These evidences should be used to organize the prescription of training. High intensity interval training may fit in futsal training with two to three working sessions during the week. In the other hand, football training is usually less intense, spending ~65 % of the time in low-to-moderate intensity (50–80 % of HRmax). For this case extensive aerobic workout combined with high intensity interval training can be the best option to apply by two weekly working sessions.

## Conclusions

The present study shows the variance of training periodization between different sexes and related team sports. Differences in workload between male and female football and futsal players were observed. Female football players spent more time in high intensity HR zones than male players suggesting that the training periodization of female players may be constrained by the fitness levels or by the type of tasks. On the contrary, such differences in the highest intensities were not found in the futsal, thus suggesting a similar type of training load imposed by the coaches. The comparison between football and futsal also revealed that the training sessions in futsal were more intense, likely following the physiological demands of the sport. These findings may help to increase the understanding about the training methodology used in different contexts and open new research opportunities to identify which tasks may control the training load for different genders.
